# Case report: Long-read sequencing identified a novel 14.9-kb deletion of the α-globin gene locus in a family with α-thalassemia in China

**DOI:** 10.3389/fgene.2023.1156071

**Published:** 2023-03-03

**Authors:** Yan Yuan, Xia Zhou, Jing Deng, Qun Zhu, Zanping Peng, Liya Chen, Ya Zou, Aiping Mao, Wanli Meng, Minhui Ma, Hongliang Wu

**Affiliations:** ^1^ Department of Medical Genetics, Yueyang Maternal and Child Health Hospital, Yueyang, China; ^2^ Berry Genomics Corporation, Beijing, China

**Keywords:** α-globin gene locus, large deletion, long-read sequencing, thalassemia, rare variant

## Abstract

**Background**: Thalassemia is a hereditary blood disease resulting from globin chain synthesis impairment because of α- and/or β-globin gene variants. α-thalassemia is characterized by non-deletional and deletional variants in the *HBA* gene locus, of which rare deletional variants are difficult to detect by conventional polymerase chain reaction (PCR)-based methods.

**Case report**: We report the case of a one-month-old boy, who and his mother had abnormal hematological parameters, while his father had normal hematology. Conventional PCR-reverse dot blot (RDB) was performed for all family members to analyze the 23 most common thalassemia variants in China, but did not identify any pathologic variants. Single-molecule real-time (SMRT) long-read sequencing (LRS) technology was then performed and identified an unreported 14.9-kb large deletion (hg38 chr16:168,803-183,737) of the α-globin gene locus, which disrupted both *HBA1* and *HBA2* genes in the proband and his mother. The exact breakpoints of the deletion were confirmed by gap-PCR and Sanger sequencing.

**Conclusion:** We have detected a novel large deletion in α-globin gene locus in China, which not only enriches the variant spectrum of thalassemia, but also demonstrates the accuracy and efficiency of LRS in detecting rare and novel deletions.

## Introduction

Thalassemia is a group of autosomal recessive hereditary blood disease resulting from globin chain synthesis impairment because of mutations in human globin genes. Severe thalassemia have serious threat to human health, causing disabilities and death (Li et al., 2014; Taher et al., 2018; Bajwa and Basit, 2022). Thalassemia gene carriers account for about 1.67% of the world’s population, but the prevalence of thalassemia has great regional differences (Weatherall, 2001; Luo et al., 2022a). Both α- and β-thalassemia occur at high frequencies throughout the tropical and subtropical regions of the world, including Southeast Aisa, the Mediterranean area, the Indian subcontinent, the Middle East, and Africa (Weatherall, 2001; Harteveld and Higgs, 2010; Piel and Weatherall, 2014). In China, the regions south of the Yangtze River have high incidence of thalassemia. Guangxi, Guangdong, Hainan, Guizhou, Yunnan, and Hunan have thalassemia carrier rates of 11%–25% (Yao et al., 2014; Ma et al., 2021; Chen P. et al., 2022a). As of 2015, there are more than 30 million thalassemia carriers in China, and out of them 300,000 have intermediate or severe thalassemia, thus it is critical to manage the health burden of thalassemia in areas prevalent with thalassemia (Huang et al., 2019).

Currently, there lacks effective approaches to cure thalassemia and carrier screening followed by prenatal diagnosis can be applied to reduce the risk of having children with severe thalassemia (Traeger-Synodinos et al., 2015; Vrettou et al., 2018). In addition, thalassemia newborn screening with hemoglobin test followed by genetic screening is an effective approach to detect thalassemia and allow for early access to specialty care. Thus, it is essential to perform genetic testing to comprehensively identify thalassemia variants. There are several types of thalassemia variants, generally including single-nucleotide variations (SNVs), indels, copy number variants including duplications and deletions. α-thalassemia variants are primarily large deletions and β-thalassemia variants are primarily SNVs/indels (Khatri et al., 2021; Luo et al., 2022b). At present, the thalassemia carrier screening strategy in China mainly focus on 23 most common variants, which include three deletions (--^SEA^, -α^4.2^, -α^3.7^), three SNVs in *HBA1/2*, and 17 SNVs/indels in *HBB* (He et al., 2014). In clinical study, occasionally the genotypes identified by conventional PCR-based methods are not well correlated well with the phenotypes, indicating the possibility of rare variations, which are beyond the detecting range of conventional methods (Aliyeva et al., 2018; Minaidou et al., 2022).

In recent years, single-molecule real-time (SMRT) long-read sequencing (LRS) technology has been used for genetic analysis of thalassemia and hemoglobin (Hb) variants (Xu et al., 2020; Liang et al., 2021; Rangan et al., 2021). Taking advantage of long reads, LRS technology can directly detect both common and rare SNVs/indels, as well as large deletions in globin genes. In this study, we identified a novel 14.9-kb deletion in the α-globin gene locus, and the exact breakpoints of the deletion by LRS technology.

### Case presentation

**FIGURE 1:**
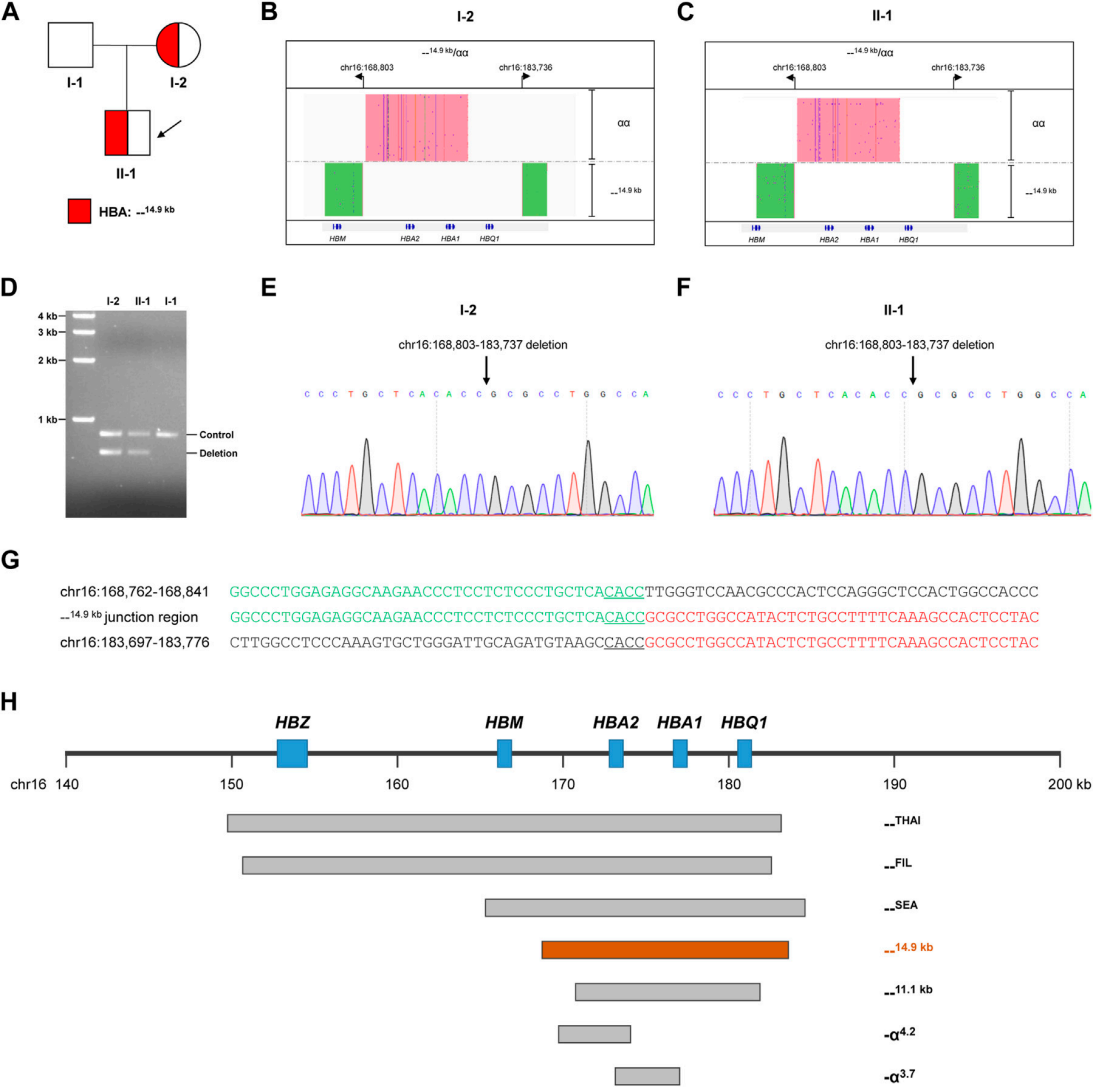
Thalassemia genetic testing by LRS technology. **(A)** Pedigree of the family. **(B, C)** Integrative Genomics Viewer plot displayed the CCS reads of SMRT sequencing for the proband (Ⅱ-1) and mother (I-2). The pink area showed the αα allele, and the green area showed the other allele with 14.9-kb deletion. **(D)** Validation of the large deletion by gap-PCR. The primers were designed flanking hg38 chr16:168803-183737 to detect the 14.9-kb deletion. **(E,F)** Confirmation of the exact breakpoints of deletions by Sanger sequencing for the proband (II-1) and mother (I-2). **(G)** Sequence of breakpoint junctions of the 14.9-kb deletion. Reference sequences encompassing the breakpoints were shown above and below the sequences with deletion. The sequences with microhomology in the breakpoint junctions were underlined. **(H)** Display of the 14.9-kb deletion in related to other recurrent deletions in the α-globin gene cluster.

The proband was a one-month-old boy with abnormal hematological parameters, which were firstly identified during thalassemia newborn screening. Hb analysis of the dried blood sample was performed by capillary electrophoresis (CE) (Capillary 2 Flex Piercing; Sebia), which showed the level of Hb Bart’s was 3.4%. Complete blood count (CBC) and Hb analysis were routinely tested for thalassemia screening in the hospital. CBC were performed with an automated hematology analyzer (Sysmex XT 1800i; Sysmex Corporation). The proband had normal white blood cell (WBC) count, red blood cell (RBC) count, platelet (PLT) count, and Hb level (Table 1). However, he showed decreased level of hematocrit (HCT) (35.4%, normal range 36%–50%), mean corpuscular volume (MCV) (79.7 fL, normal range ≥ 82 fL), mean corpuscular hemoglobin (MCH) (24.3 pg, normal range ≥ 27 pg), mean corpuscular hemoglobin concentration (MCHC) (305 g/L, normal range 310–370 g/L), indicating microcytic hypochromic anemia (Table 1). CBC and Hb analysis were also performed for his parents (Figure 1A). While the father had normal hematological parameters, the mother had decreased level of MCV (73.3 fL), MCH (23.6 pg), MCHC (307 g/L), which also indicated microcytic hypochromic anemia (Table 1). Also, the mother had decreased Hb A2 level of 1.6% (normal range 2.5%–3.5%), suggesting the existence of α-thalassemia. All the three family members had normal ferritin levels (normal range 12–135 ng/mL), which excluded iron deficiency anemia.

**TABLE 1:** Blood screening and thalassemia genetic analysis for the enrolled.

Case		Gender	Age	WBC (10^9^/L)	RBC (10^12^/L)	PLT (10^9^/L)	HCT (%)	MCV (fL)	MCH (pg)	MCHC (g/L)	Hb (g/L)	Hb A2 (%)	Hb Bart’s (%)	FER (ng/mL)	PCR-RDB	LRS
II-1	Proband	Male	1 m	9.49	4.44	475	35.4	79.7	24.3	305	108	-	3.4	31.07	Normal	HBA: --^14.9 kb^ Het
I-1	Father	Male	29 y	4.54	5.11	137	46.3	90.6	29.0	320	148	-	-	103.25	Normal	Normal
I-2	Mother	Female	19 y	10.67	5.46	279	41.0	73.3	23.6	307	129	1.6	-	29.23	Normal	HBA: --^14.9 kb^ Het

Because of abnormal hematological parameters in the proband and his mother, conventional PCR-RDB genetic analysis were performed for the family. Genomic DNA was extracted from 100 μL of peripheral blood samples using the DNA extraction kit (Chaozhou Hybyibio Limited Cororation, China), and PCR-RDB technology (Chaozhou Hybyibio Limited Cororation, China) were performed following the kit’s instructions for 23 most common thalassemia variants, including three deletional variants of α-thalassemia (--^SEA^, -α^3.7^, and -α^4.2^), three non-deletional variants of α-thalassemia (Hb Constant Spring (Hb CS, *HBA2*:c.427 T>C), Hb Westmead (Hb WS, *HBA2*:c.369C>G), and Hb Quong Sze (Hb QS, *HBA2*:c.377 T>C)), and 17 common β-thalassemia variants (−28 (*HBB*:c.-78A>G), −29 (*HBB*:c.-79A>G), −30 (*HBB*:c.-80 T>C), −32 (*HBB*:c.-82C>A), CD26 (*HBB*:c.79G>A), CD31 (*HBB*:c.94delC), CDs14-15 (*HBB*:c.45_46insG), CD17 (*HBB*:c.52A>T), CDs27-28 (*HBB*:c.84_85insC), CDs41-42 (*HBB*:c.124_127delTTCT), CD43 (*HBB*:c.130G>T), CDs71-72 (*HBB*:c.216_217insA), IVS-I-1 (*HBB*:c.92 + 1G > T), IVS-1-5 (*HBB*:c.92 + 5G > C), IVS-II-654 (*HBB*:c.316–197C > T), Int (*HBB*:c.2 T > G), and CAP (*HBB*:c.-50A>C). However, the PCR-RDB assay did not identify any variants (Figure 1B), which suggested that the microcytic hypochromic anemia in the proband and his mother might be caused by a rare thalassemia variant beyond the detecting scope of the PCR-RDB assay.

### Identification of a novel 14.9-kb deletion of *HBA* by LRS technology

LRS technology was then applied to identify potential rare thalassemia variants in the proband and his mother. Genomic DNA was amplified by PCR using primers covering the majority of known structural variations and SNVs/indels in the *HBA1*, *HBA2*, and *HBB* genes. Barcoded adaptors were added to the PCR products using a one-step end-repair and ligation reaction to construct the pre-libraries. These were then pooled together with equal mass and converted to the SMRTbell library using Sequel Binding and Internal Ctrl Kit 3.0 (Pacific Biosciences). The SMRTbell library was sequence by the consensus circular sequencing (CCS) mode of the Sequel II platform (Pacific Biosciences). After sequencing, subreads were converted to CCS reads, debarcoded to individual samples and aligned to genome build hg38 in the SMRT Link system (Pacific Biosciences). Structural variations were identified according to HbVar, Ithanet, and LOVD databases; and SNVs/indels were identified using FreeBayes1.3.4 (https://www.geneious.com/plugins/freebayes; Biomatters, Inc., San Diego, CA). Interestingly, LRS technology identified a novel heterozygous deletion in both the mother and proband, which completely impaired both *HBA1* and *HBA2* genes (Figures 1B, C). The exact 14.9-kb deletion regions (hg38 chr16:168803-183737) were successfully identified by bioinformatics analysis. Gap-PCR with primers designed flanking the deletion region confirmed the existence of deletion (Figure 1D). Then Sanger sequencing confirmed the exact breakpoints identified by the LRS technology (Figures 1E, F). Further analysis of the --^14.9 kb^ junction region showed the existence of 4-bp matching sequence, which suggested that a microhomology-mediated mechanism such as alternative end-joining or replication-based mechanisms might lead to the deletion (Figure 1G). The breakpoints of --^14.9 kb^ were different than the more recurrent deletions, such as --^THAI^, --^FIL^, --^SEA^, --^11.1 kb^, -α^4.2^, and -α^3.7^ (Figure 1H).

## Discussion and conclusion

To date, more than 120 α-globin gene variants have been identified in the Chinese population (http://www.genomed.zju.edu.cn/LOVD3/genes). α-thalassemia is mainly caused by the six common variants, of which --^SEA^, -α^3.7^,-α^4.2^, Hb CS and Hb QS account for about 90% in the Chinese population (Zheng et al., 2011). Therefore, the current thalassemia genetic screening strategy mainly aims at these variants. However, because of the technical limitations, rare deletions in the α-globin gene could not be identified.

LRS technology can be used to detect various types of genetic mutations, including SNVs/indels, structural variations, tandem repeats and fusion gene mutations (Ardui et al., 2018). LRS technology can perform comprehensive and accurate thalassemia genetic testing, avoiding misdiagnosis caused by traditional genetic methods. The clinical utility of LRS technology has also been extensively proven by several clinical studies (Xu et al., 2020; Liang et al., 2021; Luo et al., 2022a; Chen X. et al., 2022b; Luo et al., 2022b).

In this study, the following methods were used to screen for thalassemia. First, all family members performed routine blood screening, and the proband and his mother had low hematological parameters indicating microcytic hypochromic anemia. Subsequently, conventional thalassemia genetic analysis for 23 common thalassemia variants were carried out, and no variants were identified, which were inconsistent with the hematological analysis. Finally, a novel 14.9-kb deletion encompassing both *HBA1* and *HBA2* genes was identified by LRS technology, which was confirmed by gap-PCR and Sanger sequencing. This novel 14.9-kb deletion completely disrupted both α-globin genes, which caused α^0^ thalassemia. Heterozygous carrier of this deletion was suspected to have phenotype of α-thalassemia trait similarly as --^THAI^, --^FIL^, --^SEA^, and --^11.1 kb^ (Jia et al., 2004; Pongjantharasatien et al., 2016; Shang and Xu, 2017). Indeed, the hematological parameters of the proband and his mother showed mild microcytic hypochromic anemia, which were well correlated with the genotype. Thus, it is important to carry out further genetic analysis when the genotypes from routine genetic testing were inconsistent with the hematological phenotypes. Therefore, hematological analysis and genetic testing should be applied carefully to avoid false-negative results in thalassemia genetic testing. Our research has emphasized the importance of combining different technologies in achieving accurate diagnoses.

In conclusion, this study utilized LRS technology to identify a novel 14.9-kb deletion encompassing both *HBA1* and *HBA2* genes in a Chinese family, which led to α-thalassemia trait. This study demonstrated that LRS technology can improve the accuracy of thalassemia genetic testing and assist for better genotype-phenotype correlation.

## Data Availability

The datasets for this article are not publicly available due to concerns regarding participant/patient anonymity. Requests to access the datasets should be directed to the corresponding author.
